# Molecular cloning and characterization of a glycine-like receptor gene from the cattle tick *Rhipicephalus* (*Boophilus*) *microplus* (Acari: Ixodidae)

**DOI:** 10.1051/parasite/2014047

**Published:** 2014-09-02

**Authors:** José Miguel Flores-Fernández, Abel Gutiérrez-Ortega, Eduardo Padilla-Camberos, Rodrigo Rosario-Cruz, Rodolfo Hernández-Gutiérrez, Moisés Martínez-Velázquez

**Affiliations:** 1 Centro de Investigación y Asistencia en Tecnología y Diseño del Estado de Jalisco, AC Av. Normalistas 800, Col. Colinas de la Normal 44270 Guadalajara Jalisco México; 2 Centro Nacional de Investigaciones en Parasitología Veterinaria-INIFAP Carretera Federal Cuernavaca Cuautla No. 8534, Colonia Progreso 62550 Jiutepec Morelos México

**Keywords:** *Rhipicephalus* (*Boophilus*) *microplus*, glycine receptor, ivermectin, resistance, chloride channel

## Abstract

The cattle tick *Rhipicephalus* (*Boophilus*) *microplus* is the most economically important ectoparasite affecting the cattle industry in tropical and subtropical areas around the world. The principal method of tick control has relied mainly on the use of chemical acaricides, including ivermectin; however, cattle tick populations resistant to ivermectin have recently been reported in Brazil, Mexico, and Uruguay. Currently, the molecular basis for ivermectin susceptibility and resistance are not well understood in *R. microplus*. This prompted us to search for potential molecular targets for ivermectin. Here, we report the cloning and molecular characterization of a *R. microplus* glycine-like receptor (*RmGlyR*) gene. The characterized mRNA encodes for a 464-amino acid polypeptide, which contains features common to ligand-gated ion channels, such as a large N-terminal extracellular domain, four transmembrane domains, a large intracellular loop and a short C-terminal extracellular domain. The deduced amino acid sequence showed around 30% identity to GlyRs from some invertebrate and vertebrate organisms. The polypeptide also contains the PAR motif, which is important for forming anion channels, and a conserved glycine residue at the third transmembrane domain, which is essential for high ivermectin sensitivity. PCR analyses showed that *RmGlyR* is expressed at egg, larval and adult developmental stages. Our findings suggest that the deduced receptor is an additional molecular target to ivermectin and it might be involved in ivermectin resistance in *R. microplus*.

## Abstract (fr)

La tique des bovins *Rhipicephalus* (*Boophilus*) *microplus* est l’ectoparasite affectant l’industrie du bétail le plus important économiquement dans les zones tropicales et subtropicales du monde. La principale méthode de lutte contre les tiques s’est appuyée principalement sur l’utilisation d’acaricides chimiques, en particulier l’ivermectine ; toutefois, des populations de tiques des bovins résistantes à l’ivermectine ont été signalées récemment au Brésil, au Mexique et en Uruguay. Actuellement, la base moléculaire de la sensibilité et de la résistance à l’ivermectine n’est pas bien comprise chez *R. microplus*. Cela nous a incités à rechercher des cibles moléculaires potentielles de l’ivermectine. Dans ce travail, le clonage et la caractérisation moléculaire d’un gène homologue au récepteur à la glycine (*RmGlyR*) de *R. microplus* sont rapportés. L’ARNm caractérisé code pour un polypeptide de 464 acides aminés, qui contient des caractéristiques communes aux canaux ioniques ligand-dépendants, comme un grand domaine extracellulaire N-terminal, quatre domaines transmembranaires, une grande boucle intracellulaire et un court domaine C-terminal extracellulaire. La séquence d’acides aminés déduite a montré environ 30 % d’homologie avec des GlyRs de certains organismes invertébrés et vertébrés. Le polypeptide contient également le motif PAR, qui est important pour la formation de canaux d’anions, et un résidu glycine conservé dans le troisième domaine transmembranaire, qui est essentiel pour une sensibilité élevée à l’ivermectine. Des analyses PCR ont montré que *RmGlyR* est exprimé dans l’œuf, les larves et les stades de développement des adultes. Les résultats obtenus suggèrent que le récepteur décrit est une cible moléculaire supplémentaire de l’ivermectine et qu’il pourrait être impliqué dans la résistance à l’ivermectine chez *R. microplus*.

## Introduction

The cattle tick *Rhipicephalus* (*Boophilus*) *microplus* is the most economically important ectoparasite affecting the cattle industry in tropical and subtropical areas around the world [16]. Reduction in weight gain and milk production, and skin damage, besides transmission of pathogens causing bovine babesiosis and anaplasmosis, result in substantial economic losses for cattle producers. Currently, the principal method of tick control is the application of chemical acaricides; however, their indiscriminate and intensive use has led to the development of tick populations resistant to organophosphates, pyrethroids, amidines, and macrocyclic lactones, which represents a worldwide drawback for successful tick control [6, 23, 24, 27, 30]. Ivermectin (IVM) is one of the macrocyclic lactones most used for tick control, and resistance to IVM has been shown in some countries such as Brazil and Mexico, and recently an incipient resistance in Uruguay [6, 15, 23, 26]. Despite the fact that *R. microplus* is a parasite with known IVM susceptibility, the molecular basis for this susceptibility, as well as the mechanisms involved in IVM resistance, are currently poorly understood. IVM activates gamma-aminobutyric acid-gated (GABA), glutamate-gated (GluCl), and glycine-gated (GlyR) chloride ion channels in nerve and muscle cells in arthropods and nematodes, causing paralysis of peripheral motor function, inhibition of feeding and reproduction and, ultimately, death [7, 30, 37, 38]. These effects are believed to occur in *R. microplus* ticks exposed to IVM, presumably via the same receptors. However, until recently, just a GABA receptor and a GluCl channel have been characterized in this tick [8, 14]. The discovery of molecular targets for IVM provides an opportunity to understand its biological effects better, to determine the molecular mechanisms that lead to acaricide resistance, and for the identification of novel acaricide agents. In this study, we report the molecular cloning and characterization of a glycine-like receptor gene, and its expression in different development stages in *R. microplus* ticks.

## Materials and methods

### Ticks and tissues

The *R. microplus* Media Joya strain, which is susceptible to all major classes of acaricides, was obtained from CENID-PAVET-INIFAP, located in the State of Morelos, Mexico. Ovarian and intestinal tissues were obtained by dissection from engorged female ticks as described by Tsuda et al. [36].

### RNA extraction

Total RNA was extracted from different development stages; eggs, larvae, and adult tissues, using TRIZOL reagent (Invitrogen, Carlsbad, CA, USA). cDNA was synthesized from 4 μg of RNA using the SuperScript^®^ III First-Strand Synthesis System (Invitrogen, Carlsbad, CA, USA) according to the manufacturer′s instructions.

### 5′ Rapid amplification of cDNA ends (RACE)

A TC2637 EST sequence was obtained from a database of cDNAs expressed in *R. microplus* (BmiGI database, http://compbio.dfci.harvard.edu/tgi/cgi-bin/tgi/gimain.pl?gudb=b_microplus). To characterize the 5′ end sequence of the *GlyR* receptor, specific primers for the sequence were designed (GSP1*GlyR* 5′-CAAGGTGAACGTGGCATTGA-3′ and GSP2*GlyR* 5′-GAACACGCCTGTGTCGATAGC-3′), which were used in conjunction with the AAP primer provided by the 5′ RACE system (Invitrogen, Carlsbad, CA, USA) as outlined by the manufacturer. PCR conditions were as follows: 94 °C for 5 min, 35 cycles of 94 °C for 30 s, 55 °C for 30 s, and 72 °C for 90 s, and then 72 °C for 10 min.

### Cloning and sequencing

The putative receptor amplicon was purified and then cloned into the pCR®2.1-TOPO T/A plasmid vector (Invitrogen, Carlsbad, CA, USA) which was used to transform competent *Escherichia coli* TOP10 cells. *E. coli* was then incubated overnight at 37 °C on LB plates containing ampicillin and X-gal. Five white colonies were isolated and cultured overnight in LB medium containing 50 μg/mL ampicillin. Plasmid DNA was extracted using the QIAprep Spin Miniprep Kit (Qiagen, Hilden, Germany) according to the manufacturer’s instructions. The positive transformants were analyzed by PCR using forward and reverse M13 primers. Once the correct clone was identified, the plasmid DNA was submitted to the Laboratorio Nacional de Biotecnología Agrícola, Médica y Ambiental (LANBAMA) of the Instituto Potosino de Investigación Científica y Tecnológica (IPICYT) (San Luis Potosí, Mexico) for sequencing. The new sequence was deposited in GenBank with accession number KJ476181.

### Polymerase chain reaction

The following primers (Integrated DNA Technologies, Inc., Coralville, IA, USA) were used: F-*RmGlyR* 5′-ATG CTT GAG CAG TAC GAC AA-3′ and R-*RmGlyR* 5′-TAT CAA GTG GTA GCC ATT CTG G-3′ using cDNA from eggs, larvae, and adult tissues as a template. PCR conditions were 94 °C for 5 min followed by 35 cycles, each consisting of denaturation at 94 °C for 30 s, annealing at 54 °C for 30 s, and extension at 72 °C for 45 s. A final extension step at 72 °C for 10 min was included. The amplified products were run on 1% agarose gels and were analyzed on a UV transilluminator (Bio-Rad Laboratories, Philadelphia, PA, USA). Control amplification was carried out using the following primers: F-5′-GAC GCA GAT CATGTT CGA GA-3′ and R-5′-ACA GGT CCT TAC GGA TGT CG-3′, designed from *R. microplus actin* (GenBank accession number AY255624.1). PCR conditions were 94 °C for 5 min followed by 35 cycles, each consisting of denaturation at 94 °C for 30 s, annealing at 60 °C for 30 s, and extension at 72 °C for 45 s, with a final extension step at 72 °C for 7 min. The results are expressed as a ratio of the density of the *RmGlyR* product to the density of the *actin* products from the same template.

### Bioinformatics analysis

Primers were designed with Primer3 software v. 4.00 [http://primer3.wi.mit.edu/; 32]. The search for similarity among sequences was conducted using the BLAST service from the National Center for Biotechnology Information (NCBI, USA) [http://blast.ncbi.nlm.nih.gov/Blast.cgi; 1]. The nucleotide to amino acid sequence translation was done using the Translate Tool [http://web.expasy.org/traslate/; 9]. The signal peptide sequence was predicted using the SignalP 4.1 server [http://www.cbs.dtu.dk/services/SignalP/; 28]. Membrane spanning domains were predicted by the TMHMM Server at the Center for Biological Sequence Analysis, Technical University of Denmark, DTU [http://www.cbs.dtu.dk/services/TMHMM/; 18, 19]. The two-dimensional representation was made using TMRPres2D [http://bioinformatics.biol.uoa.gr/TMRPres2D/; 35]. The molecular weight and theoretical isoelectric point were predicted by the ExPASy ProtParam tool [http://web.expasy.org/protparam/; 10]. Multiple alignments of the nucleotide and amino acid sequences were performed using the multiple alignment program ClustalW [http://www.genome.jp/tools/clustalw/; 19].

## Results

### Identification of the *RmGlyR* gene and full-length cDNA cloning

**Figure 1.**
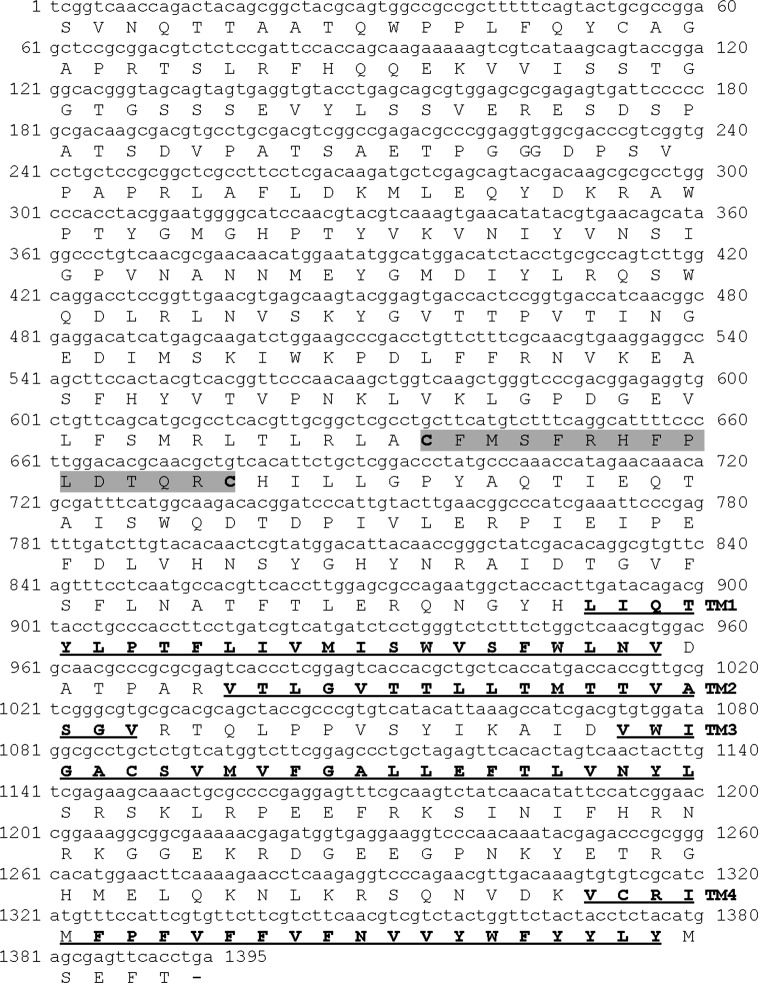
Nucleotide and deduced amino acid sequence of the putative *GlyR* channel from *R. microplus*. The cysteines forming the Cys-loop are highlighted in gray shading and the putative membrane domains (TM1–TM4) are underlined in bold. The hyphen indicates the stop codon.

**Figure 2.**
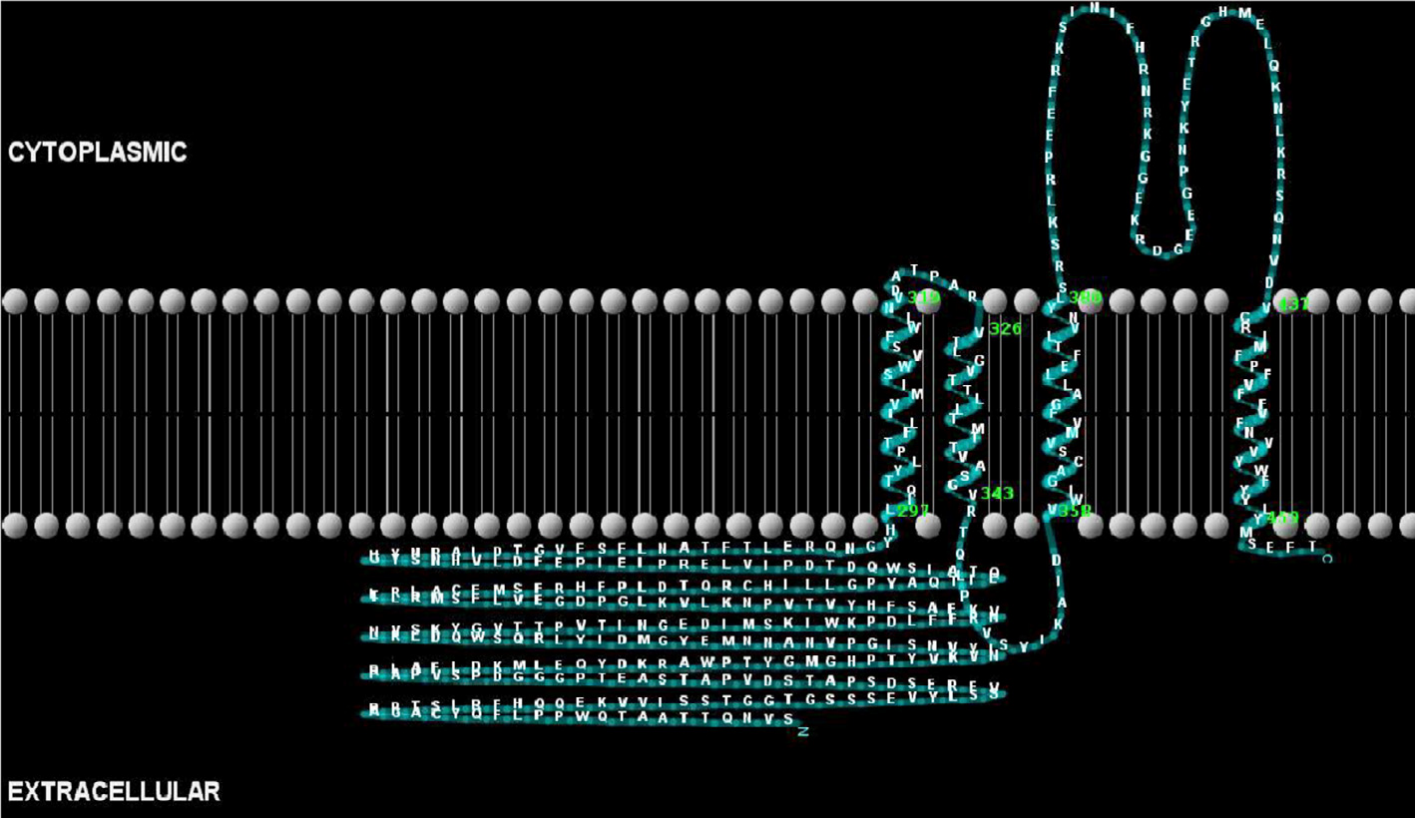
Two-dimensional representation of the putative GlyR channel from *R. microplus* showing the extracellular, transmembrane and intracellular domains.

The molecular basis for IVM susceptibility and resistance are not well understood in *R. microplus* ticks. This prompted us to search for novel molecular targets for IVM. Guerrero et al. [11] deposited a number of EST sequences with similarity to genes with possible roles in response to acaricide exposure or development of acaricide resistance. We focused on the EST TC2637 (GenBank accession no. CK184967), given that tentatively encodes for a chloride channel, a known target for IVM, and proceeded to further characterization. Based on the TC2637 sequence, we designed specific oligonucleotides GSP1*GlyR* and GSP2*GlyR* and used the 5′ RACE system to characterize the missing 5′ end of the *GlyR* sequence. Once we found the correct ORF and the deduced stop codon position, we designed PCR primers for the amplification of the respective full-length cDNA, which was cloned and sequenced (KJ476181). Sequence analysis showed that the flanked region has an ORF 1392 bp that encodes for a 464-amino acid polypeptide (Fig. 1), which contains features common to ligand-gated ion channels, such as a large amino-terminal extracellular domain, four transmembrane domains and a large intracellular loop (Fig. 2), with a predicted molecular mass of 52.85 kDa and a pI of 8.13. The deduced polypeptide appears to have no signal peptide.

**Figure 3.**
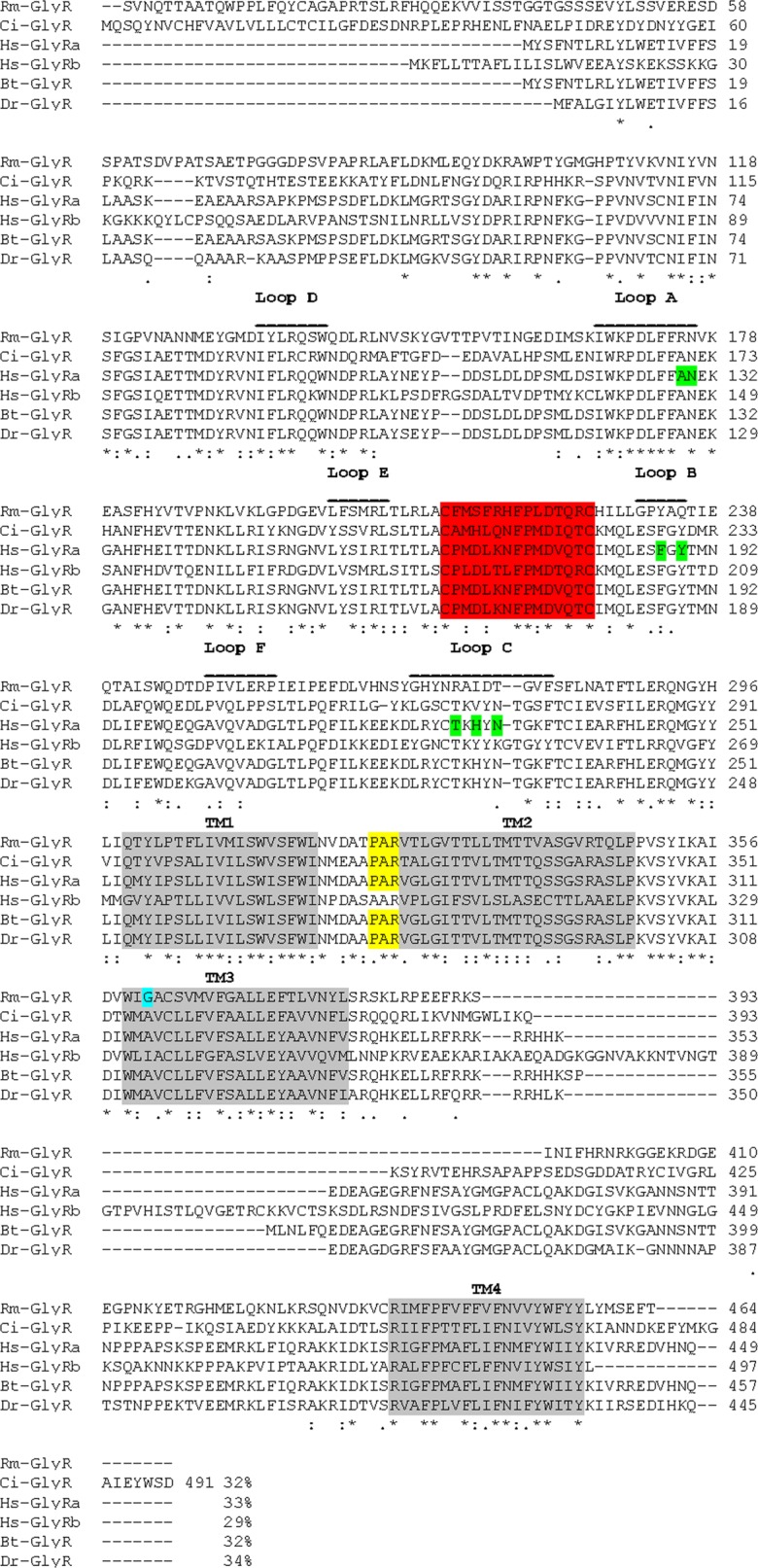
Alignment of the deduced amino acid sequence of GlyR from *R. microplus* (KJ476181), GlyR from *C. Intestinalis* (BAI66458), GlyRa (NP_000162) and GlyRb (NP_000815) from *H. sapiens*, GlyR from *B. taurus* (NP_776746), and GlyR from *D. rerio* (NP_571477). The cysteines forming the Cys-loop are highlighted by red shading and the putative membrane domains (TM1–TM4) are highlighted by gray shading. The PAR residues are highlighted in yellow shading. The glycine residue marked in turquoise likely confers upon the receptor high sensitivity to ivermectin. The approximate locations of the glycine binding domains are labeled as A-F. Known residues in human GlyRa that play a role in glycine binding are highlighted in green [22].

BLAST analysis showed 32%, 34%, and 32% identities among *RmGlyR* and GlyRs from *Ciona intestinalis*, *Danio rerio* and *Bos taurus*, respectively, and 33% and 29% with the subunits a and b of GlyR from *Homo sapiens*, respectively. All compared amino acid sequences contain four transmembrane domains and two cysteine residues that form the characteristic Cys-loop (Fig. 3). The sequences also share the PAR signature at the end of the first intracellular loop (Fig. 3, shaded in yellow). This signature is associated with most anionic Cys-loop (ligand-gated ion channels) LGICs, more specifically in those labeled as alpha subunits.

### Expression pattern of *RmGlyR* in the life cycle of *R. microplus*

**Figure 4.**
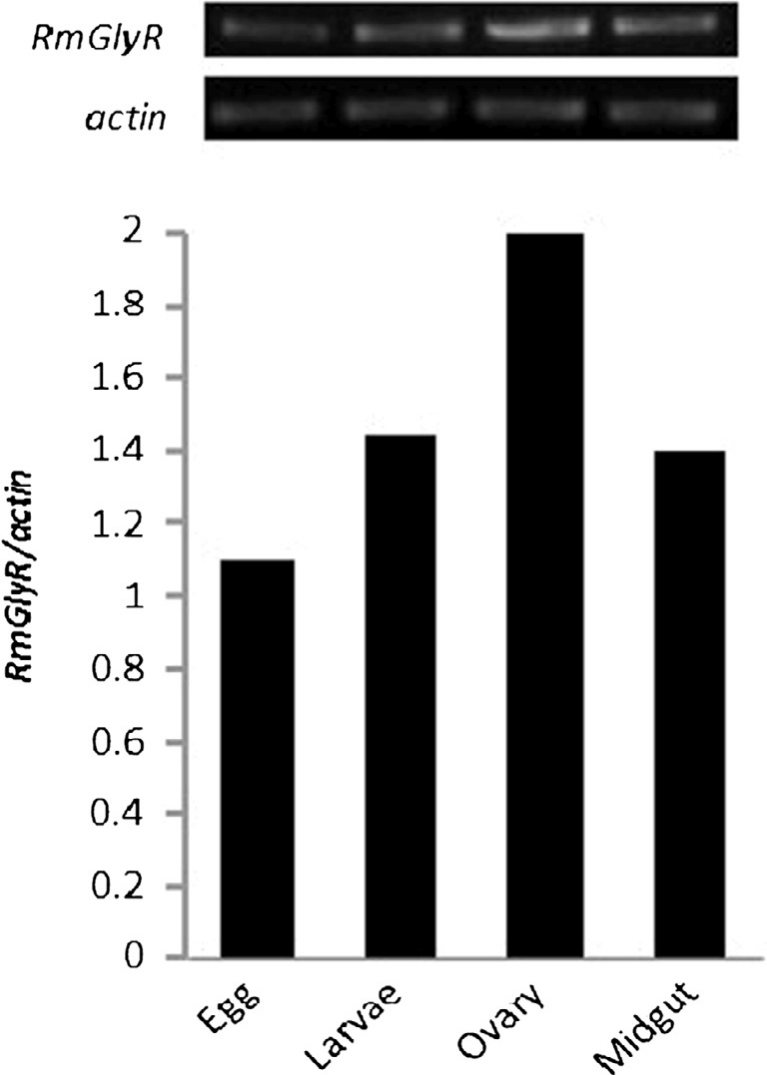
Expression of the *RmGlyR* gene in different stages of *R. microplus*. RT-PCR reactions were performed using total RNA from egg, larvae and adult tissues (ovary and midgut). Tick *actin* gene was used as a control. The data are expressed as a density ratio between *RmGlyR* and *actin* products.

To determine the expression profiles of the *RmGlyR* gene, total RNA was extracted from tissues of different developmental stages, including eggs, larvae, and adult. RT-PCRs were performed using the primers F-*RmGlyR* and R-*RmGlyR*. The expression levels of *RmGlyR* were normalized by the *actin* levels, as mentioned before. The highest expression of *RmGlyR* was observed in adult ovary tissue, whereas at the egg stage the expression level was lower (Fig. 4).

## Discussion

Acaricide resistance in *R. microplus* is routinely determined using bioassays. These methods, however, are time-consuming to perform and, depending on the acaricide mode of action, can yield relatively poor sensitivity [31]. The use of molecular methods offers researchers the opportunity to find new molecular targets for acaricide susceptibility and resistance detection, as well as the chance to discover new drugs against ticks. Macrocyclic lactones such as IVM play a major role in controlling *R. microplus* ticks. Nonetheless, the molecular target sites for IVM have not yet been elucidated and characterized at the molecular level in this parasite. IVM activates GABA and glutamate-gated chloride channels [38], both of which belong to the Cys-loop LGICs, which constitute a family of pentameric channels defined by a fixed-length cysteine loop in the N-terminal extracellular domain and four transmembrane (TM) regions. These Cys-loop receptors mediate the synaptic transmission of nerve impulses [13]. In other arthropods and nematodes, IVM activates GluCls at nanomolar concentrations. Higher (micromolar) concentrations of IVM also activate or modulate vertebrate Cys-loop receptors, including the excitatory nicotinic and the inhibitory GABA type-A and glycine receptors (GlyRs) [21, 34]. Hope et al. [14] characterized the first GABA receptor from *R. microplus* that belongs to the Cys-loop LGIC family, and identified a mutation that causes a codon change from threonine to leucine, which confers resistance to dieldrin. Later, Gassel et al. [8] reported another member of the LGIC family, a glutamate-gated (GluCl) chloride channel. In this work we report a glycine-like receptor, the newest member of the LGIC family in *R. microplus*. *RmGlyR* contains a large N-terminal extracellular domain (ECD), four TM regions (TM1–TM4), a large intracellular loop between the regions TM3 and TM4, and a short extracellular C-terminal domain. In the ECD the ligand binding occurs, whereas the TM2 region cooperates to form the ion channel pore, and the large intracellular loop is important for protein-protein interactions involved in trafficking, clustering, intracellular modulation, and related processes [3, 17, 22]. Glycine-gated channels had been found only in vertebrates but recently GlyRs have been identified and characterized in some invertebrates such as *Ciona intestinalis* and *Hydra vulgaris* [25, 29, 33]. Additionally, the sequencing of the starlet sea anemone (*Nematostella vectensis*) genome predicted the existence of a glycine receptor [2].

The *RmGlyR* transcript was expressed throughout development in *R. microplus*, with the highest expression level seen in adult ovary tissue. Similar expression patterns have been observed in other Cys-loop LGICSs from *Drosophila melanogaster* and *Tetranychus urticae* [4, 5].

**Figure 5.**
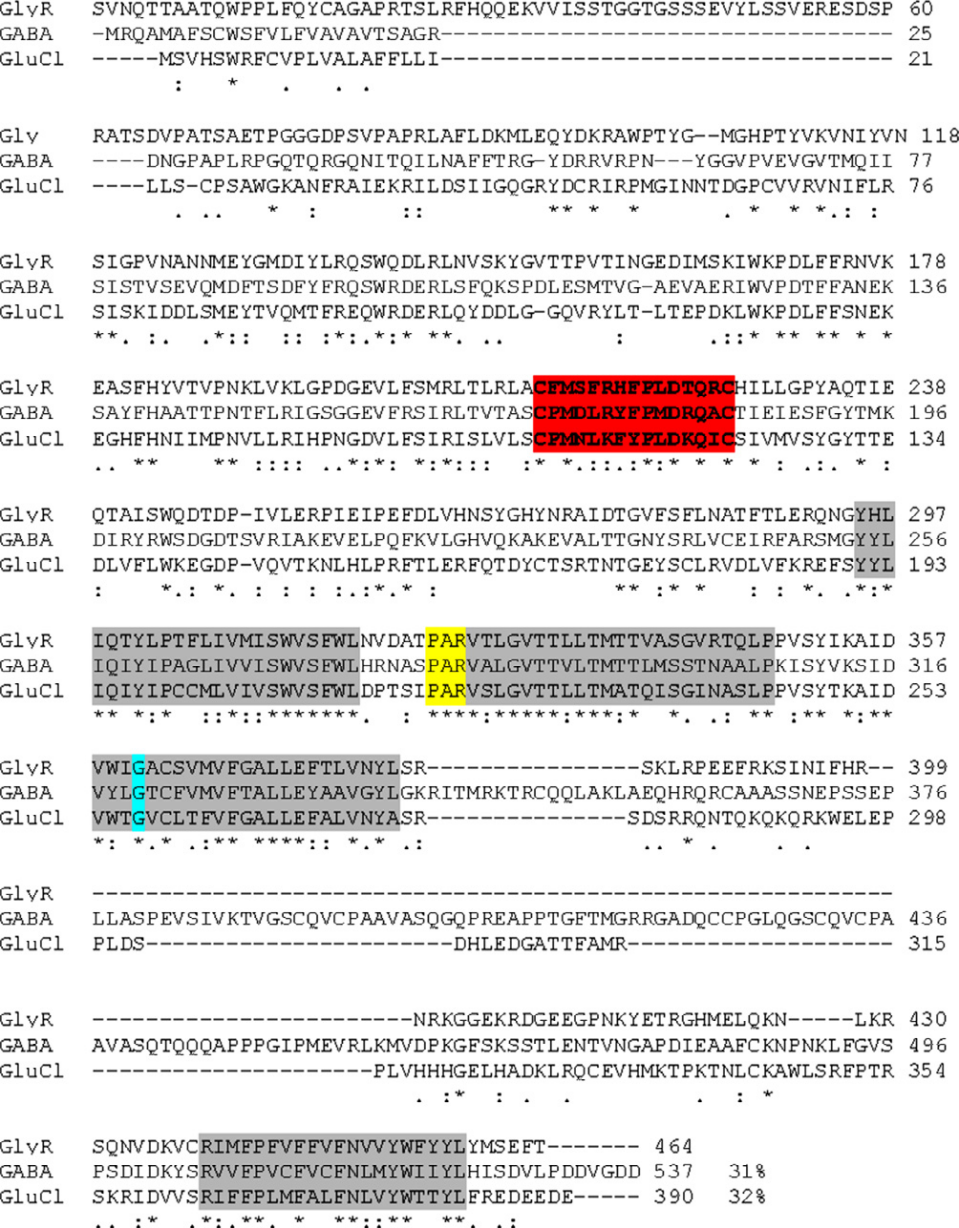
Alignment of the deduced amino acid sequences of GlyR (KJ476181), GluCl (AHE41097) and GABA (AHE41094) receptors from *R. microplus*. The cysteines forming the Cys-loop are highlighted by red shading; the putative transmembrane domains (TM1–TM4) are shaded in gray. The PAR residues are highlighted in yellow. The glycine residue marked in turquoise likely confers upon the receptors sensitivity to ivermectin.

Hibbs and Gouaux [12] recently solved the crystal structure of the *Caenorhabditis elegans* αGluCl complexed with IVM. The IVM binding site is formed at the interface of two adjacent subunits in the transmembrane domain of the receptor. Given the high structural similarity among GluCls and GlyRs, it is possible that IVM binds in a common location in the GluCl and GlyR. Lynagh and Lynch [20] showed that a glycine residue at the TM3 transmembrane domain location is essential for high IVM sensitivity in both glycine- and glutamate-gated Cys-loop receptors. We compared the amino acid sequences of the three LGICs reported so far in *R. microplus* and surprisingly detected a conserved TM3-Glycine residue in the three receptors (Fig. 5, shaded in turquoise). The presence of this glycine residue would predict high IVM sensitivity of these LGICs, in contrast to the human GlyRs (Fig. 3). We also found that these receptors possess the PAR motif before the transmembrane region 2 (TM2), which is important for forming anion channels (Fig. 5) [5]. The results from the protein sequence homology analyses and other bioinformatic predictions indicate that we have identified the *R. microplus* gene ortholog for GlyR. Taken together, these findings point out GABA, GluCl, and GlyR as being the molecular targets for IVM in *R. microplus*. This may well explain the susceptibility of the tick to this class of acaricide, although we do not discard the fact that more targeted LGICs may exist. The functional characterization of these receptors is required to enhance our understanding of the mechanism of action of IVM, which will facilitate the management of macrocyclic lactone resistance in *R. microplus*.

## Conclusions

In summary, we report the molecular characterization of a glycine-like receptor from *R. microplus*. To our knowledge, this is the first report of a GlyR from ticks. Electrophysiological studies are underway to determine GlyR regulation by the agonist glycine and the acaricide IVM. Finally, the identification of molecular targets for IVM helps develop the foundation for the study of molecular mechanisms that lead to acaricide resistance and for the development of novel acaricides.
